# Bibliometric and Visualized Analyses of Research Studies on Different Analgesics in the Treatment of Orthopedic Postoperative Pain

**DOI:** 10.1155/2022/6835219

**Published:** 2022-02-24

**Authors:** Yunzhong Cheng, Honghao Yang, Li Guan, Yong Hai, Aixing Pan

**Affiliations:** ^1^Department of Orthopedic Surgery, Beijing Chao-Yang Hospital, Capital Medical University, Beijing 100020, China; ^2^Department of Neurobiology, School of Basic Medical Sciences, Beijing Key Laboratory of Neural Regeneration and Repair, Beijing Institute for Brain Disorders, Capital Medical University, Beijing 100020, China

## Abstract

**Background:**

Pain following orthopedic surgery has always been a critical issue, which caused great distress to the patients. Analgesics in the treatment of postoperative pain following orthopedic surgery have aroused great attention from scholars, and numerous studies have been published in recent years. Bibliometrics could assist scholars in understanding the scope of research topics better, identifying research focuses and key literature, and analyzing the development and trend of analgesics in the treatment of postoperative pain following orthopedic surgery.

**Methods:**

Literature data were retrieved from the Science Citation Index Expanded (SCI-E) of Web of Science (WOS) Core collection database. The articles from 1992 to December 2021 on analgesics in the treatment of postoperative pain following orthopedic surgery were recruited. The citation reports including the publication numbers, h-index, total citations, and average citations in terms of authors, organizations, and countries were obtained. Top 20 research directions, funds, and journals with the most publications were charted. The co-authorship relations in the analysis units of authors, organizations, and countries were analyzed by the online bibliometric tool and VOSviewer software. The author's keywords co-occurrence overlay map was visualized by the VOSviewer software.

**Results:**

A total of 406 articles were retrieved from 1992 to December 4th, 2021, with 11,655 times cited, average citations of 28.57 per item, and an h-index of 55. The most high-yield publication year, authors, organizations, countries, research directions, funds, and journals were 2020 (*n* = 887), Ilfeld BM from University of California San Diego (*n* = 7), University of California System (*n* = 21), the USA (*n* = 178), Anesthesiology (*n* = 161), National Institutes of Health (NIH), USA, and United States Department of Health Human Services (*n* = 12), and Anesthesia and Analgesia (*n* = 29), respectively. Similarly, co-authoring analysis of publications regarding on different analgesics showed that the authors and countries with the most co-authorship strength were Carr Daniel B (total link strength = 6) and the USA (total link strength = 30), respectively. The highest occurrence keywords were “postoperative pain” with 135 occurrences (total link strength = 784). The future research hotspots might be “acute pain,” “outcomes,” “oxycodone,” “total hip,” “replacement,” and “United States.”

**Conclusion:**

Analgesics in the treatment of postoperative pain following orthopedic surgery can be observed in this study by employing the online bibliometric tool and VOSviewer software, which established the relationship between the units of analysis. It can provide a meaningful resource with detailed information for orthopedic surgeons who would like to understand the trend in this field better. They can also benefit from the emphasis on citation count to carry out high-level research in the future.

## 1. Introduction

Postoperative pain has always been a problem plaguing clinical treatment. In addition to the pain of the incision, many other factors can also cause pain, especially following the orthopedic operations, such as periosteal irritation, swelling of the affected limb, increased bone fascia tension, postoperative compression bandaging, and external fixation [1]. More importantly, pain after orthopedic surgery becomes more common and more severe. What is worse, pain can increase cortisol, blood sugar, tea phenolamine in the body, and tissue metabolism, which is not conducive to wound healing. Therefore, the management of postoperative analgesia following orthopedic surgery is very important [2].

There are more than 53 million records and 1.18 billion cited references in the Science Citation Index Expanded (SCI-E) of Web of Science (WOS) Core collection database, which is an important resource for scientific statistics and evaluation [3]. More frequent citations of the article play a more important role in the field [4]. A bibliometric analysis and visualization tool can effectively assess the thematic development of structural contents and help readers understand a field comprehensively [5].

However, no bibliometric research on different analgesics in the treatment of postoperative pain following orthopedic surgery has been performed. This study aims to outline the intellectual connections within the dynamic changing of scientific knowledge in orthopedic postoperative analgesia using the WOS Core Collection database and the VOSviewer software.

## 2. Methods

The literature data were retrieved through SCI-E of WOS Core Collection database, which is widely applied in bibliometric research using an advanced search strategy. The search query was “(((((TS = (Orthopedic OR Orthopedic Procedure OR Orthopedic Surgery OR Orthopedic Surgical Procedure)) AND TS = (Postoperative OR Postoperative Periods)) AND TS = (Pain OR Physical Suffering OR Ache)) AND TS = (Analgesics OR anodyne OR Analgesic Drugs OR Analgesic OR Analgesic Agents OR Antinociceptive Agents)) AND LA = (English) AND DT = (Article).” Timespan = all years. All articles were evaluated by two independent reviewers in order to confirm their relevance. Full records of all articles were searched on December 4th, 2021.

The trends of publications and citations were charted annually. The distribution of the bibliographic records per year in different countries was also obtained. The top 20 most cited articles were recorded and analyzed by the following information: first author, article title, journals of publication, year of publication, total number of citations, and average citations. The records, h-index, total citations, and average citations in terms of authors, organizations, and countries were tabulated directly. The top 20 research directions, funds, and journals with the most publications were charted. The co-authorship relations in the analysis units of authors, organizations, and countries were mapped by the online bibliometric tool (https://bibliometric.com/) and VOSviewer 1.6.11 software (Nees Jan van Eck and Ludo Waltman, 2019). The author's keywords co-occurrence overlay map was implemented by VOSviewer, setting the minimum occurrences of a keyword to 5 times.

## 3. Results

### 3.1. Publication Outlines

**Figure 1:**
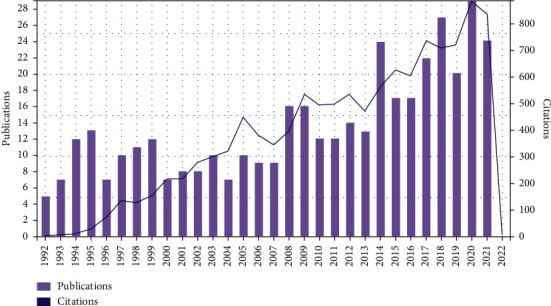
Annual publications (column chart) and sum of times cited per year (curve line) on analgesics in the treatment of orthopedic postoperative pain from 1992 to 2021.

A total of 406 articles were retrieved in the SCI-E of WOS Core Collection database from 1992 to December 4th, 2021, with 11,655 times cited, average citations of 28.57 per item, and an h-index of 55.  Figure 1 shows the annual publications and sum of times cited per year on analgesics in the treatment of postoperative pain following orthopedic surgery. The first article was published in 1992, and the year with the most publications (*n* = 29) was 2020. The citation started in 1992, and the year with the most times cited was 2020 (*n* = 887). The results showed a fluctuating increase year by year.

**Figure 2:**
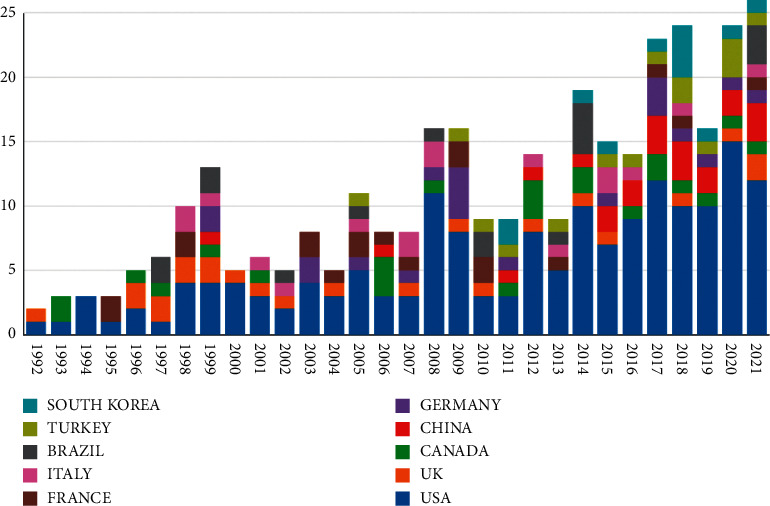
The distribution of the bibliographic records per year of the top 10 countries on analgesics in the treatment of orthopedic postoperative pain.

The USA had contributed 178 articles (43.842%) at the top. Canada was the second contributing country with 25 articles (6.157%), followed by England with 22 articles (5.419%) and France and Germany both with 20 articles (4.926%). China only ranked 9th with 13 articles (3.186%). Only two countries contributed articles in 1992. However, more and more countries published articles yearly, and the number of involved countries increased to ten in 2021. The USA dominates in this field almost every year. The distribution of the bibliographic records each year of the top 10 countries on analgesics in the treatment of postoperative pain following orthopedic surgery is shown in Figure 2.

### 3.2. Top 20 Most Cited Articles on Different Analgesics

This search collected a total of 406 articles between 1992 and 2021 from WOS. The top 20 most cited articles are given in Table 1, including first author, article title, journals of publication, year of publication, total number of citations, and average citations. The total citations of the top 20 articles ranged from 101 to 1013. The average citations of the top 20 articles ranged from 4.21 to 40.52. The most cited article had 1013 citations and was published in 1997 by Collins et al. [6], followed by Chung et al. [7] with 271 citations in 1997 and Sinatra et al. [8] with 252 citations in 2005. The first two published articles were by Laitinen and Nuutinen [9] and Baker [10] in February 1992, and the most recent articles were published in December 2021 by De Biase et al. [11] and Rajput et al. [12].

### 3.3. Contribution of Authors, Organizations, and Countries

There were 1,961 authors, 745 organizations, and 46 countries contributing to this field.  Table 2 provides the top 5 high-yield authors (Ilfeld Brian M, Lauretti Gabriela Rocha, Carr Daniel B, Liu Spencer S, and Capdevila Xavier [13–17]), organizations (University of California System, Pennsylvania Commonwealth System of Higher Education Pcshe, Universidade De São Paulo, University of Pennsylvania, and Cleveland Clinic Foundation), and countries (the USA, Canada, England, France, and Germany), with the corresponding records, h-index, total citations, and average citations.

### 3.4. Contribution of Research Directions, Funds, and Journals

**Figure 3:**
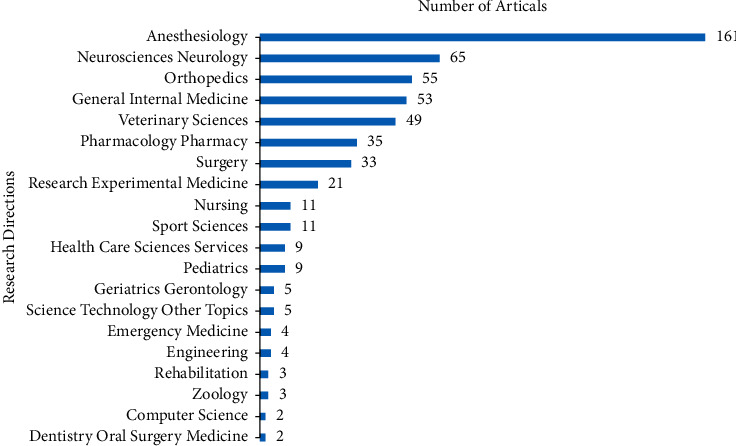
Top 20 research directions with the most publications on analgesics in the treatment of orthopedic postoperative pain.

**Figure 4:**
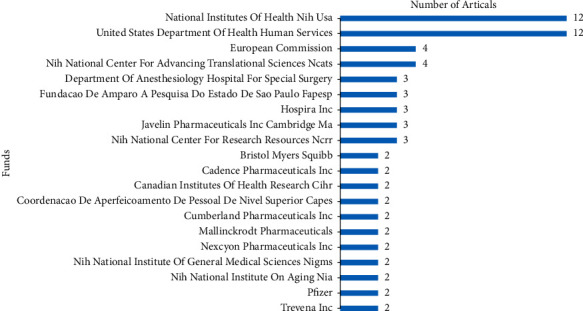
Top 20 funds for the most publications on analgesics in the treatment of orthopedic postoperative pain.

**Figure 5:**
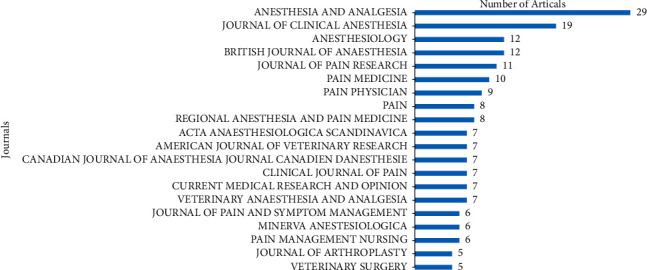
Top 20 journals with the most publications on analgesics in the treatment of orthopedic postoperative pain.

There were 37 research directions, 154 funds, and 174 journals contributing to this topic.  Figure 3 shows the top 20 high-yield research directions, the top is Anesthesiology with 161 publications, followed by Neurosciences Neurology with 65 publications and Orthopedics with 55 publications. In the top 20 high-yield funds, National Institutes of Health (NIH), USA, and United States Department of Health Human Services were tied for first place with 12 publications, followed by European Commission and NIH National Center for Advancing Translational Sciences (NCATS) both with 4 publications (Figure 4).  Figure 5 shows the top 20 high-yield journals. Anesthesia and Analgesia had contributed 29 articles (7.108%) at the top. Journal of Clinical Anesthesia was the second contributing journal with 19 articles (4.657%), followed by Anesthesiology and British Journal of Anesthesia both with 12 articles (2.941%).

### 3.5. Co-Authoring Analysis of Publications regarding Different analgesics

**Figure 6:**
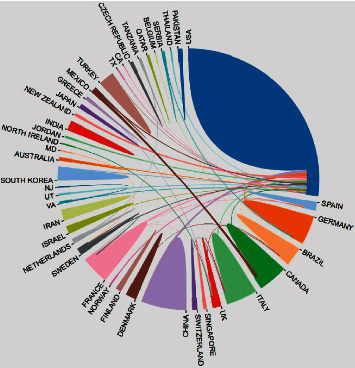
Country co-authoring analysis of publications regarding analgesics in the treatment of orthopedic postoperative pain.

The authors with the most co-authorship strength were Carr Daniel B, Daniels et al. [18], Viscusi et al. [19], and Lauretti and Reis [20] (Table 3). The strongest collaborative country was the USA with 4,411 citations (total link strength = 30), followed by France with 599 citations (total link strength = 9), Canada with 997 citations (total link strength = 8), Germany with 530 citations (total link strength = 8), and Sweden with 279 citations (total link strength = 7) (Figure 6).

### 3.6. Keywords Co-Occurrence

**Figure 7:**
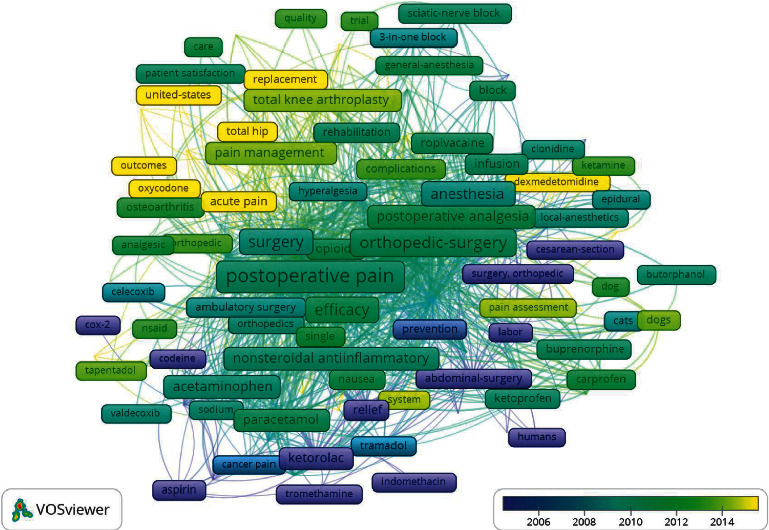
Keywords co-occurrence overlay mapping on analgesics in the treatment of orthopedic postoperative pain. Each frame indicates a keyword. The rainbow color marks the average publication year from violet (further year) to yellow (recent year) in the range of spectrum. The larger scale of a keyword is according to the higher frequency, while the closer distance between the two keywords represents the stronger co-occurrence.

The overlay visualization of the top 162 co-occurrence keywords is shown in Figure 7. The highest occurrence keyword was “postoperative pain” with 135 occurrences (total link strength = 784), followed by “morphine” with 91 occurrences (total link strength = 616), “orthopedic-surgery” with 91 occurrences (total link strength = 582), “analgesia” with 85 occurrences (total link strength = 550), and “pain” with 83 occurrences (total link strength = 522). The most recent keywords were “acute pain,” “outcomes,” “oxycodone,” “total hip,” “replacement,” and “United States.”

## 4. Discussion

Bibliometrics mainly collects bibliographic databases and bibliometric features and uses mathematical and statistical methods to qualitatively and quantitatively analyze the relevant information of the literature, such as the distribution of countries, authors, journals, institutions, and funds. It also helps researchers grasp the development trend of this field intuitively and quickly [21, 22]. Using visual analysis software to analyze the literature further, researchers can find current research hotspots and directions in this field. These methodologies have been widely applied in orthopedic research studies [23, 24].

### 4.1. Analysis on Publication Outlines

The popularity of a specific topic can be reflected by the number of publications. The study on analgesics in the treatment of postoperative pain following orthopedic surgery was initially published in 1992. The number of articles published fluctuated around ten from 1992 to 2007 and increased significantly from 2008 to 2021. Meanwhile, the quality of a specific topic can be judged by the number of citations [25]. There was an exponential growth in the citation times from 1992 to 2021. From Figure 1, we can learn that the future trend on analgesics in treating orthopedic postoperative pain looks promising.

As shown in Figure 2, the number of articles published from the USA is dominant in this field, followed by the UK and Canada. The article initially published from China was in 1999. The number of articles published from China is far less than that in the USA, which may be due to less attention paid to this field by Chinese scholars.

### 4.2. Analysis on Top 20 Most Cited Articles on Different Analgesics

Citation analysis is a systematic method to evaluate the influence of scientific research [26]. An article with more frequent citation can be recognized as more influential in the specific field [4]. As given in Table 1, the total citation of each article on different analgesics was more than 100. The most cited article was a clinical trial by Collins et al. in 1997 in Pain [6], focusing on visual analogue scales (VAS). The results indicate that if a patient records a VAS score over 30 mm at baseline, they would probably have recorded at least moderate pain on a 4-point categorical scale. After that, the VAS score has been more widely used by many scholars, which may explain why this article was ranked at the top with 1013 citations.

The second most cited article was a retrospective review by Chung et al. in 1997 in Anesthesia and Analgesia [7], focusing on the pattern of pain in ambulatory surgical patients and determining those factors that predict postoperative pain. The final results of the article showed that anesthesiologists give adequate analgesia by taking into consideration the body mass index of the patient, the duration of anesthesia, and the type of surgery. Better methods of postoperative pain treatment, such as using NSAIDs, regional techniques, and multimodal analgesia techniques, are needed. The third most cited article was a clinical trial by Sinatra et al. in 2005 in Anesthesiology [8], focusing on pain intensity, pain relief, and morphine use. The results indicate that intravenous acetaminophen, 1 g, administration over a 24-h period in patients with moderate to severe pain after orthopedic surgery could be well tolerated with rapid and effective analgesia.

The latest literature was a randomized controlled trial by Marino et al. in 2009 in Journal of Bone and Joint Surgery, paying attention to continuous lumbar plexus block for postoperative pain control after total hip arthroplasty. The conclusion was that continuous lumbar plexus and femoral blocks significantly reduce the need for opioids and decrease related side effects [27]. Another literature was a retrospective research by Hebl JR in 2008 in Regional Anesthesia and Pain Medicine, suggesting that a preemptive multimodal pathway featuring peripheral nerve block improves perioperative outcomes after major orthopedic surgery [28].

### 4.3. Analysis on Contribution of Authors, Organizations, and Countries

H-index refers to *h* articles in the literature that have been cited at least *h* times by other scholars, which is a measure to evaluate an author or country by the number of academic output and the index of the academic output level [29, 30]. The total number of references cited refers to the number of times a document has been cited in a certain period, an important indicator for evaluating individual national influence [31].

Among the top five high-yield authors, three come from the USA, Ilfeld BM from University of California San Diego, Carr DB from Tufts University, and Liu SS from Virginia Mason Medical Center. Similarly, four were in the USA among the top five high-yield organizations, including University of California System, Pennsylvania Commonwealth System of Higher Education Pcshe, University of Pennsylvania, and Cleveland Clinic Foundation. This may explain why the USA was ranked at the top one with a total of 178 records, which is far more than that in other countries (Table 2).

### 4.4. Analysis on Contribution of Research Directions, Funds, and Journals

As shown in Figures 3–5, anesthesiology, neurosciences, neurology, and orthopedics were the hot research directions, which will help orthopedic physicians to catch the right directions better.

In addition, National Institutes of Health (NIH), USA, and United States Department of Health Human Services were the most high-yield funds. Both of them belong to the USA. This was a good reason to explain why the USA was dominant in this field.

Identifying the dominant journals in a specific topic can help scholars construct scientific achievement. Anesthesia and Analgesia, Journal of Clinical Anesthesia, Anesthesiology, and British Journal of Anesthesia were the most high-yield journals. Paying more attention to high-yield journals can assist scholars in accessing the most authoritative knowledge framework and the orientation of manuscript submitting. The publishers of these journals belong to the USA, while the rest one is from the UK. Researchers may benefit from this important information and realize the deficiencies when high-level articles appear [32].

### 4.5. Analysis on Co-Authoring Analysis of Publications regarding Different Analgesics

VOSviewer was used for cooperation network analysis of authors, organizations, and countries [33]. Carr DB, Daniels SE, and Singla N were the authors with the most link strength. However, the most link strength was only six, which indicated that the cooperation between the authors was less (Table 3).

As shown in Figure 6, the strongest collaborative countries were the USA with 4,411 citations (total link strength = 30), followed by France with 599 citations (total link strength = 9) and Canada with 997 citations (total link strength = 8). The rest were mainly from developed countries. The result showed that the USA have the most cooperation with other countries.

### 4.6. Analysis on Keywords Co-Occurrence

VOSviewer was also used to generate a keyword co-occurrence map [34]. The highest occurrence keyword was “postoperative pain” with 135 occurrences (total link strength = 784), followed by “morphine” with 91 occurrences (total link strength = 616) and “orthopedic-surgery” with 91 occurrences (total link strength = 582). The most recent keyword was “acute pain,” “outcomes,” “oxycodone,” “total hip,” “replacement,” and “United States,” which indicated that these keywords might be the future research hotspots (Figure 7).

## 5. Limitations

As all we know, bibliometric analysis has been widely used to measure the impact of articles in recent years. However, there are still some limitations to this method. First and foremost, we only used the core collection of WOS to search literature, which is a single database. The more databases we use, the more information we can get and analyze. Other databases such as InCites and MEDLINE should be considered in the future. Second, the main language of WOS is English. Articles written in other languages are excluded, which means some relevant articles be omitted. Third, the citation number of each study is time-dependent. For different time points to search the articles, different citations may be obtained. However, the trend of citation number of each study is nearly the same.

This study has several advantages despite the limitations mentioned. It is the first study using the bibliometric method to search and identify literature on analgesics in the treatment of postoperative pain following orthopedic surgery, which has attracted increasing attention in recent years. Most importantly, our study provides valuable information for orthopedic surgeons and researchers in this field.

## 6. Conclusion

To conclude, we researched and analyzed the literature information regarding authors, organizations, countries, research directions, funds, and journals and analyzed the thematic development and future research hotspots. Our research observes the raising concern on analgesics in the treatment of orthopedic postoperative pain in recent years. Anesthesia and Analgesia, Journal of Clinical Anesthesia, Anesthesiology, and British Journal of Anesthesia are the most influential journals. The future research hotspots might be “acute pain,” “outcomes,” “oxycodone,” “total hip,” “replacement,” and “United States.”

## Figures and Tables

**Table 1:** Top 20 most cited articles on analgesics in the treatment of orthopedic postoperative pain.

First author	Article title	Journal	Publication year	Total citations	Average citations
Collins SL	The visual analogue pain intensity scale: What is moderate pain in millimetres?	Pain	1997	1013	40.52
Chung F	Postoperative pain in ambulatory surgery	Anesthesia and Analgesia	1997	271	10.84
Sinatra RS	Efficacy and safety of single and repeated administration of 1 g intravenous acetaminophen injection (paracetamol) for pain management after major orthopedic surgery	Anesthesiology	2005	252	14.82
Feldt KS	Treatment of pain in cognitively impaired compared with cognitively intact older patients with hip-fracture	Journal of the American Geriatrics Society	1998	192	8.00
Lascelles BDX	Efficacy and kinetics of carprofen, administered preoperatively or postoperatively, for the prevention of pain in dogs undergoing ovariohysterectomy	Veterinary Surgery	1998	173	7.21
Reuben SS	Postoperative analgesic effects of celecoxib or rofecoxib after spinal fusion surgery	Anesthesia and Analgesia	2000	169	7.68
Briggs M	A descriptive study of the use of visual analogue scales and verbal rating scales for the assessment of postoperative pain in orthopedic patients	Journal of Pain and Symptom Management	1999	168	7.3
Rapp SE	Acute pain management in patients with prior opioid consumption: a case-controlled retrospective review	Pain	1995	161	5.96
Moore A	Deriving dichotomous outcome measures from continuous data in randomised controlled trials of analgesics	Pain	1996	158	6.08
Hebl JR	A preemptive multimodal pathway featuring peripheral nerve block improves perioperative outcomes after major orthopedic surgery	Regional Anesthesia and Pain Medicine	2008	153	10.93
White PF	The use of a continuous popliteal sciatic nerve block after surgery involving the foot and ankle: Does it improve the quality of recovery?	Anesthesia and Analgesia	2003	134	7.05
Liu SS	Patient-controlled epidural analgesia with bupivacaine and fentanyl on hospital wards: prospective experience with 1,030 surgical patients	Anesthesiology	1998	129	5.38
Gwirtz KH	The safety and efficacy of intrathecal opioid analgesia for acute postoperative pain: Seven years' experience with 5969 surgical patients at Indiana University Hospital	Anesthesia and Analgesia	1999	125	5.43
Dohoo SE	Postoperative use of analgesics in dogs and cats by Canadian veterinarians	Canadian Veterinary Journal-Revue Veterinaire Canadienne	1996	125	4.81
Gimbel JS	Efficacy and tolerability of celecoxib versus hydrocodone/acetaminophen in the treatment of pain after ambulatory orthopedic surgery in adults	Clinical Therapeutics	2001	114	5.43
Hernandez-Palazon J	Intravenous administration of propacetamol reduces morphine consumption after spinal fusion surgery	Anesthesia and Analgesia	2001	109	5.19
Collins SL	Seeking a simple measure of analgesia for mega-trials: Is a single global assessment good enough?	Pain	2001	108	5.14
Marino J	Continuous lumbar plexus block for postoperative pain control after total hip arthroplasty a randomized controlled trial	Journal of Bone and Joint Surgery-American Volume	2009	105	8.08
Grisneaux E	Comparison of ketoprofen and carprofen administered prior to orthopedic surgery for control of postoperative pain in dogs	Journal of the American Veterinary Medical Association	1999	101	4.39
Peduto VA	Efficacy of propacetamol in the treatment of postoperative pain: morphine-sparing effect in orthopedic surgery	Acta Anaesthesiologica Scandinavica	1998	101	4.21

**Table 2:** The top five high-yield countries, organizations, and authors on analgesics in the treatment of orthopedic postoperative pain from 1985 to 2021.

Category	Rank	Items	Records	H-index	Total citations	Average citations
Author	1	Ilfeld BM, University of California San Diego	7	5	226	32.29
	2	Lauretti GR, University of São Paulo	6	4	171	28.5
	3	Carr DB, Tufts University	5	5	77	15.4
	3	Liu SS, Virginia Mason Medical Center	5	5	220	44
	4	Capdevila X, CHU de Montpellier Anesthesiol and Crit Care Dept	4	4	197	49.25
Organization	1	University of California System	21	12	602	28.67
	2	Pennsylvania Commonwealth System of Higher Education Pcshe	9	5	160	17.78
	2	Universidade De Sao Paulo	9	5	206	22.89
	3	University of Pennsylvania	8	6	124	15.5
	4	Cleveland Clinic Foundation	7	3	120	17.14
Country	1	USA	178	36	4777	26.84
	2	Canada	25	18	1272	50.88
	3	England	22	16	2064	93.82
	4	France	20	13	667	33.35
	4	Germany	20	13	531	26.55

**Table 3:** Co-author analysis of publications regarding analgesics in the treatment of orthopedic postoperative pain.

Author	Number of articles	Citations	Total link strength
Carr DB	5	77	6
Daniels SE	4	124	6
Singla N	4	83	6
Lauretti GR	5	171	4
Reis MP	4	167	4
Turan A	4	60	3
Ilfeld BM	4	82	2
Minkowitz HS	4	169	1

## Data Availability

The data used to support the findings of this study are available from the corresponding author upon request.
